# Population genetic diversity and hybrid detection in captive zebras

**DOI:** 10.1038/srep13171

**Published:** 2015-08-21

**Authors:** Hideyuki Ito, Tanya Langenhorst, Rob Ogden, Miho Inoue-Murayama

**Affiliations:** 1 Wildlife Research Center, Kyoto University, 2-24 Tanaka-Sekiden-cho, Sakyo, Kyoto, 606-8203, Japan; 2 Kyoto City Zoo, Okazaki Park, Okazaki Houshoji-cho, Sakyo, Kyoto, 606-8333, Japan; 3 Marwell Wildlife, Colden Common, Winchester, Hants SO21 1JH, United Kingdom; 4 Royal Zoological Society of Scotland, Royal Zoological Society of Scotland, 134 Corstorphine Road, Edinburgh, Scotland, EH12 6TS, United Kingdom; 5 Wildlife Genome Collaborative Research Group, National Institute for Environmental Studies, 16-2 Onogawa, Tsukuba, Ibaraki, 305-8506, Japan

## Abstract

Zebras are members of the horse family. There are three species of zebras: the plains zebra *Equus quagga*, the Grevy’s zebra *E. grevyi* and the mountain zebra *E. zebra*. The Grevy’s zebra and the mountain zebra are endangered, and hybridization between the Grevy’s zebra and the plains zebra has been documented, leading to a requirement for conservation genetic management within and between the species. We characterized 28 microsatellite markers in Grevy’s zebra and assessed cross-amplification in plains zebra and two of its subspecies, as well as mountain zebra. A range of standard indices were employed to examine population genetic diversity and hybrid populations between Grevy’s and plains zebra were simulated to investigate subspecies and hybrid detection. Microsatellite marker polymorphism was conserved across species with sufficient variation to enable individual identification in all populations. Comparative diversity estimates indicated greater genetic variation in plains zebra and its subspecies than Grevy’s zebra, despite potential ascertainment bias. Species and subspecies differentiation were clearly demonstrated and F1 and F2 hybrids were correctly identified. These findings provide insights into captive population genetic diversity in zebras and support the use of these markers for identifying hybrids, including the known hybrid issue in the endangered Grevy’s zebra.

Zebras belong to the taxonomic family of horses (Equidae), which is comprised of a single genus, *Equus*. Zebras are native to Africa and are characterized by their distinctive black and white striped coat. They occur in a variety of habitats, including grasslands, savannas, woodlands, thorny scrublands, mountains, and coastal hills^1^,^2^,^3^. There are three species of zebra: the plains zebra (*Equus quagga*), the mountain zebra (*E. zebra*) and the Grevy’s zebra (*E. grevyi*). The plains zebra has five extant subspecies: Burchell’s zebra *E. q. burchelli*, Grant’s zebra *E. q. boehmi*, Selous’ zebra *E. q. borensis*, Chapman’s zebra *E. q. chapmani*, and Crawshay’s zebra *E. q. crawshayi*^2^. The mountain zebra has two subspecies: Cape mountain zebra *E. z. zebra* and Hartmann’s mountain zebra *E. z. hartmannae*^1^. The plains zebra and the mountain zebra belong to the subgenus *Hippotigris*, but Grevy’s zebra is the sole species of subgenus *Dolichohippus*. While plains zebras are plentiful, various anthropogenic factors (over hunting, competition with livestock, habitat loss, etc.) have had a severe impact on Grevy’s zebra and mountain zebra populations, which are now listed as Endangered or Vulnerable respectively on the IUCN red list^1^,^3^. There is relatively little information on the genetic diversity of these species to support conservation management in the wild or in captivity; the development and application of molecular genetic tools is therefore an important consideration.

Analysis of genetic structure using microsatellite markers and mtDNA has been reported in mountain zebra^4^ and plains zebra^5^. However, in Grevy’s zebra, although a limited number of microsatellite markers have been identified^6^, there has been no detailed analysis of genetic structure with nuclear genetic markers. Furthermore, hybridization between the Grevy’s zebra and the Grant’s zebra, a sub-species of plains zebra has been reported in the wild, resulting in fertile F1 individuals^7^. Hybridization may be a potential risk factor in the conservation of Grevy’s zebra and it is therefore essential to be able to genetically identify these admixed individuals and to investigate how these hybridized individuals affect Grevy’s zebra populations.

Microsatellite markers are one of the most popular genetic markers for conservation genetic studies^8^. However, their application to endangered species with little existing genetic information has traditionally been complicated by laborious and time-consuming work and associated high development costs. Recently, the use of next-generation sequencing techniques for microsatellite marker development has reduced time and costs, providing a powerful tool for genetic studies in non-model/endangered species^9^,^10^,^11^,^12^,^13^. Previously, we developed 21 microsatellite markers in the Grevy’s zebra using next-generation sequencing^14^. The aim of this study was to investigate the utility of an expanded Grevy’s zebra microsatellite marker panel to identify subspecies, detect hybrids and assess population genetic diversity in all three zebra species. To achieve this we have characterized an additional seven novel microsatellite markers, evaluated cross-species amplification in the plains and mountain zebras and conducted a series of simulation studies to determine the power of these genetic tools to detect hybridization in captive zebra populations.

## Results

We excluded 38 faecal samples that had failed to genotype at more than five loci. The numbers of samples used subsequent analysis in Grevy’s zebra, plains zebra (Grant’s zebra, Chapman’s zebra) and Hartmann’s mountain zebra were 52, 27 (15, 12) and 6 respectively.

### Validation of utility for cross-species amplification

The results of cross-species amplification for all 28 loci are shown Table 1 (for details, see Supplementary Table S1 and S2, and representative peak patterns in Supplementary Figure S1 and S2). For the estimation of population genetic indices, the plains zebra was divided into Grant’s and Chapman’s subspecies and analyzed separately. The data for mountain zebra are presented here for information, however the sample size is too small to allow accurate estimates to be obtained. Seven loci x species (sub-species) combinations showed deviations from Hardy–Weinberg Equilibrium after sequential Bonferroni correction (three loci in Grant’s zebra, two loci in Grant’s zebra, and two loci Chapman’s zebra), but no single locus showed deviation in more than one species. Allellic richness (*Ar*), the number of alleles (*Na*), observed heterozygosity (*Ho*) and expected heterozygosity (*He*) in combined all loci are indicated in Table 1 (The indices in each locus are indicated in Supplementary Table S1 and S2). Polymorphism is generally conserved across species, with 25 polymorphic markers in Grevy’s zebra (the discovery species) compared to 27 markers in plains zebra (Grant’s zebra *n* = 27; Chapman’s zebra *n* = 26) and 23 markers in mountain zebra. A greater number of private alleles was observed in plains zebra (*n* = 63) than in Grevy’s zebra (*n* = 37) or mountain zebra (*n* = 19) and allelic richness and mean heterozygosity were higher in plains zebra (and its individual subspecies) than Grevy’s zebra (Table 1).

Cumulative probability of identity (*PID*) and *PID-sib* across all loci ranged from 2.28 × 10^−14^ to 7.63 × 10^−21^ and 1.06 × 10^−6^ to 5.67 × 10^−9^ respectively, supporting the use of the marker panels for individual identification in all populations.

### Differentiation of species and sub-species

**Figure 1:**
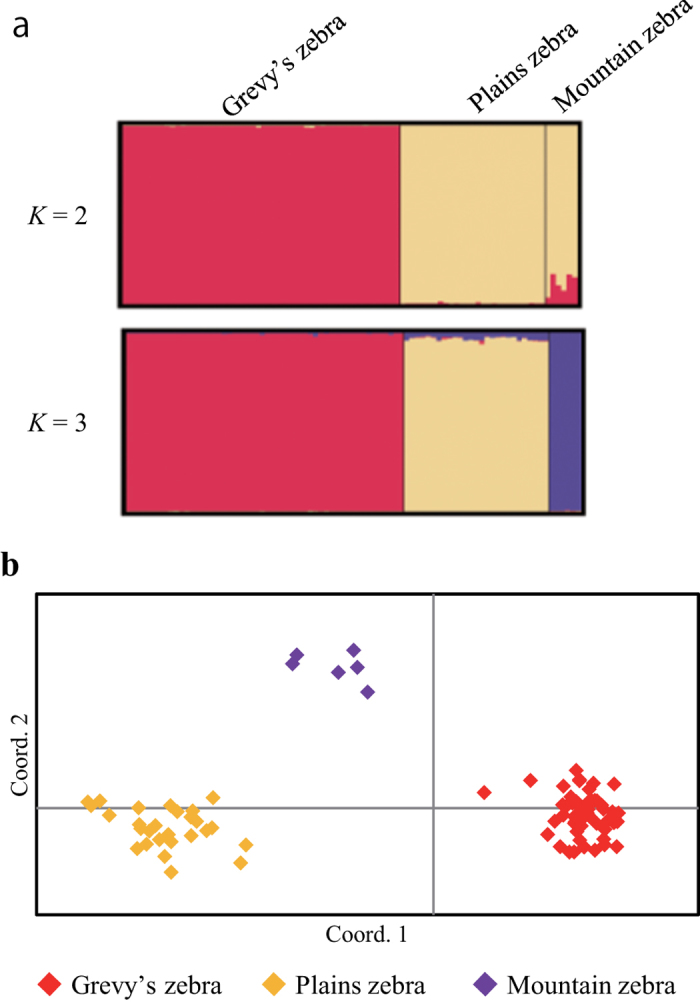
(**a**) Bayesian analysis of the genetic structure showing differentiation of three zebra species based on 28 microsatellite loci. (**b**) First and second components of a principal coordinate analysis of 28 microsatellite loci in three zebra species.

The clustering of three zebra species observed in the STRUCTURE analysis (Fig. 1a) and principal component analysis (Fig. 1b) demonstrates clear separation of the three species using microsatellite data. In STRUCTURE analysis, greatest support was found for K = 2 clusters, resulting in the separation of Grevy’s zebra from the other two zebra species (Fig. 1a); at *K* = 3 (Fig. 1a), plains zebra and mountain zebra were subsequently differentiated unambiguously. The confidence in individual assignment was high, with the Grevy’s zebra assigned to cluster I with average proportion of membership *Q*_*I*_ = 99.6%, plains zebra assigned to cluster II with average proportion of membership *Q*_*II*_ = 99.4%, and mountains zebra assigned to cluster III with average proportion of membership *Q*_*III*_ = 99.5%. Additionally, PCoA separated the three species clearly. Percentages of variation explained by the first 2 axes were 22.4% and 7.0%, respectively.

**Figure 2:**
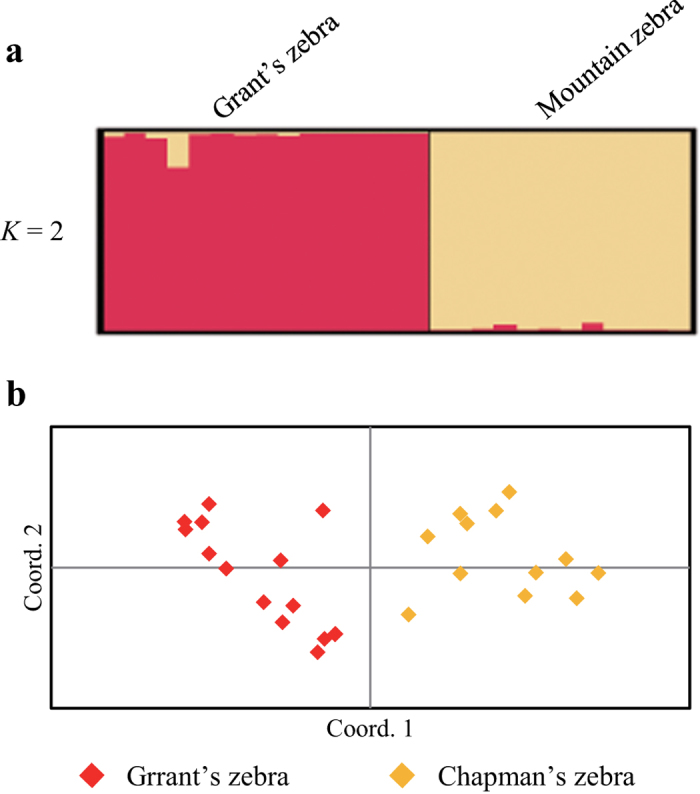
(**a**) Bayesian analysis of the genetic structure showing differentiation of two Plains zebra subspecies (Grant’s and Chapman’s) based on 28 microsatellite loci. (**b**) First and second components of a principal coordinate analysis of 28 microsatellite loci in plains zebra explained 16.6% and 10.8% of total variance, respectively.

The results of structure analysis and PCoA in the two sub-species of plains zebra show separation ofthe Grant’s zebra from Chapman’s zebra (Fig. 2). At *K* = 2, average proportion of cluster membership in both Grant’s zebra *Q*_*I*_ = 98.2%, and Chapman’s zebra *Q*_*II*_ = 99.1% was high.

### Detection of hybridized individuals

**Figure 3:**
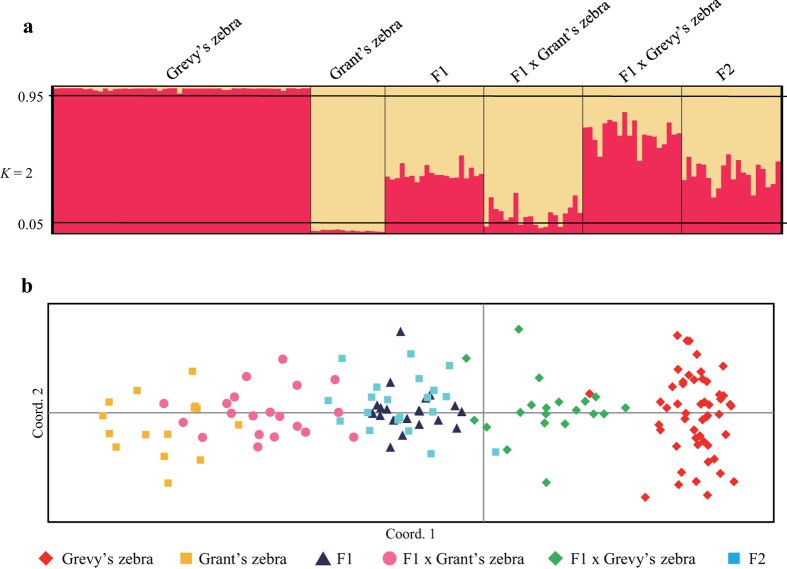
(**a**) Bayesian analysis of the genetic structure of the Grevy’s zebra, Grant’s zebra and their hybrids (F1, F2, and back cross) based on 28 microsatellite loci. (**b**) First and second components of a principal coordinate analysis of 28 microsatellite loci in the Grevy’s zebra, Grant’s zebra and hybridization (F1, F2, and back cross). Percentages of variation explained by the first 2 axes were 18.4% and 3.8%, respectively.

The results of STRUCTURE analysis and PCoA in the six populations comprised of two pure species (Grevy’s zebra and Grant’s zebra) and four hybridized populations (Grevy’s zebra x Grant’s zebra = F1; F1 × F1 = F2; F1 × Grevy’s zebra backcross = BxGy, F1 × Grant’s zebra = BxGt) indicate a sharp distinction between the pure species and two of the hybridized populations (F1 and F2 populations). As expected,the backcrossed populations were less clearly differentiated, with the F1 x Grant’s zebra backcross partly overlapping with Grant’s zebra (Fig. 3). STRUCTURE results for assignment to Grevy’s zebra for actual and simulated individuals were as follows: For Grevy’s zebra *Qi* (population average) and *qi* (individual range) scores were: Grevy’s zebra 0.986 (0.954–0.991); Grant’s zebra, 0.016 (0.009–0.024); simulated F1s, 0.404 (0.347–0.531); F1 x Grevy’s zebra backcrosses, 0.681 (0.493–0.826); F1 x Grant’s zebra backcrosses, 0.125 (0.034–0.275); and in simulated F2s, 0.406 (0.243–0.539).

## Discussion

This study has demonstrated the utility of a 28 marker microsatellite panel for assessing subspecies identity and hybridization in zebras, including endangered species where hybridization is recognized as a potential conservation issue. All markers successfully were cross-amplified from Grevy’s zebra to plains and mountain zebra with high levels of conserved polymorphism. While direct comparison of population genetic diversity among subspecies is potentially affected by ascertainment bias, given that the markers were isolated in Grevy’s zebra, it is interesting to note that indices of population genetic diversity for plains zebra (and its two subspecies) are higher, suggesting comparatively less genetic diversity exists in the captive Grevy’s zebra population. No comparative data exists for captive population founder size in these two species, but as the number of wild plains zebra (~600,000) far exceeds that of Grevy’s zebra (~2,500)^32^, this result is perhaps not surprising, and indicates that an assessment of the wild Grevy’s zebra population would be useful to evaluate the proportion of species genetic diversity that is represented in the captive conservation breeding programme.

The power of the microsatellite panels for individual DNA profiling and differentiation of siblings indicated that these markers should be suitable for the identification of all individuals in captivity and the wild. Overall the results demonstrate that these novel microsatellite markers are useful for the analysis of genetic diversity and identification of individuals in three zebra species, although the numbers of mountain zebra should be increased to strengthen these findings.

Species identification using STRUCTURE was accurate and unambiguous, with PCoA analysis also showing clear differentiation of species clusters. Between subspecies of plains zebra, all individuals were also assigned correctly but with a lower level of confidence. However the proportion of membership (*qi*) of all individuals was higher than 0.8 (minimum 0.83) used as a target for admixed individuals^15^,^16^,^17^. This result demonstrates that captive Grant’s zebra and Chapman’s zebra can be readily separated using DNA markers. While allopatric distribution of these two sub-species minimizes the risk of hybridization in the wild, they are commonly kept in captivity and not managed by studbook, so there is a risk that these sub-species might have hybridized in the past. The lack of studbook data for Grant’s zebra and Chapman’s zebra also means that we cannot quantify the proportion of captive subspecies diversity sampled in this study. As samples were derived from relatively few zoos, further sampling would be recommended to increase confidence in these findings.

The index of admixture in Bayesian analysis has been frequently used to identify individual introgression^15^,^18^,^19^,^20^, and has been used in the detection of hybridized individuals by defining threshold values of *qi*^17^. In order to increase the exclusion of potential hybrids, some studies have used stringent *qi* values (>0.95) to define non-hybridized individuals^21^,^22^ and we adopted this high *qi* threshold value (>0.95) to confidently identify pure individuals in this study. Assignment of simulated hybrid individuals to their hybrid category (F1, F2, BxGy, BxGt) deviated from expectations (0.5, 0.5, 0.75 and 0.25, respectively) with lower average assignment to the Grevy’s zebra genetic lineage. This deviation may be due to the fact that a greater number of species-specific alleles were observed in Grant’s zebra than Grevy’s zebra, skewing the assignment of simulated individuals.

Pure species (Grevy’s zebra and Grant’s zebra) could be clearly separated from the simulated F1 and F2 populations. Cordingley *et al.*^7^ reported observing F1 individuals derived from natural matings between male Grevy’s zebra and female Grant’s zebra in Kenya, with the F1 individuals subsequently staying in the Grant’s zebra group. Therefore, to conserve the endangered Grevy’s zebra, it is important to be able to discriminate between Grevy’s zebra and hybridized individuals. This study demonstrates that microsatellite markers can distinguish Grevy’s zebra from hybridized individuals in captive populations. In addition to their usefulness in captive population genetic management in all three species, these markers are expected to distinguish hybrids between these taxa in wild populations. However as the population allele frequencies generated within this study are unlikely to accurately reflect the situation in the wild due to founder effects and drift, further analysis using wild population samples from potential hybrid zones is strongly recommended.

## Methods

### Samples

This study was conducted in strict accordance with the guidelines for the ethics of animal research by the Wildlife Research Center of Kyoto University. The sampling and methods were approved by each zoo providing samples and the Wildlife Research Center of Kyoto University. We obtained blood, muscle, hairs and faeces from Grevy’s zebra (*n* = 60), plains Zebra (*n* = 53) (Grant’s Zebra, *n* = 33; Chapman’s Zebra, *n* = 20) and Hartmann’s mountain zebra (*n* = 10). All individuals were kept in zoos in Japan or the United Kingdom. Invasive sampling was minimized, with blood samples collected as bi-products during health examination and hairs samples (ca. 10 hairs) plucked by keepers and muscle samples obtained post mortem. DNA was extracted from whole blood, muscle and hair using the QIAGEN DNeasy Blood and Tissue Kit (QIAGEN), and from faeces using the QIAGEN DNeasy Stool Kit (QIAGEN).

### Development of microsatellite markers

In addition to the 21 Grevy’s zebra microsatellite markers previously published^14^, a further seven markers were used in this study, developed at the same time using the same method. DNA was extracted from blood of male Grevy’s zebra using QIAGEN DNeasy Blood and Tissue Kit (QIAGEN). After checking the quality of genomic DNA by resolution on a 0.5% agarose gel and spectrophotometry (Nanodrop, USA), 500 ng of the genomic DNA was nebulized at 0.24 MPa for 1 min, and purified using the MinElute PCR Purification kit (QIAGEN). The fragments were end-repaired, A-tailed and ligated to the Rapid Library Adapter with RL Ligase (Roche). Short fragments were removed using AMPure XP beads, and the quality and quantity of the library were assessed using Agilent 2100 Bioanalyser (Agilent). Library fragments were mixed with capture beads and clonally amplified through emulsion PCR using the GS-Junior Titanium emPCR kit (Roche). Captured fragments were enriched and annealed with sequencing primers and sequenced using GS-Junior bench-top sequencer (Roche). We obtained 92,254 reads, and the reads containing microsatellite were screened by MSATCOMMANDER^23^. Repeats including 2-6 nucleotides repeat were searched for with the following settings: more than seven di-repeats and more than four repeats for the other repeat types. Among reads containing microsatellite, sixty-six primers were designed using the PRIMER3^24^. And these primer pairs were tested for amplification and polymorphism in the Grevy’s zebra. We selected 21 microsatellite markers developed previously^14^, novel four polymorphic markers and three monomorphic markers in this study. All 28 markers were tested for cross-species amplification in plains and mountain zebra and the additional seven markers also examined for the first time in Grevy’s zebra.

PCR amplifications were performed by modified protocol of the Qiagen Multiplex PCR Kit (Qiagen) in a final volume of 10 μl, which contained 20 ng of extracted DNA, 2.5 μl Multiplex PCR Master Mix, 400 μM of each dNTP, 0.4 μM of forward (fluorescently labeled) and reverse primers. In faecal samples, instead of 20 ng DNA, 2 μl of extracted DNA solution and 0.1 μg of T4 Gene 32 Protein (Nippon Gene) were added. Blood, hair and tissue sample PCR conditions consisted of an initial denaturation at 95 °C for 15 min, followed by 30 cycles of denaturation at 94 °C for 30 s, annealing at 60 °C for 1 min 30 s, extension at 72 °C for 1 min, and final extension at 60 °C for 30 min. In faecal samples, the PCR conditions consisted of an initial denaturation at 95 °C for 15 min, followed by 15 cycles of denaturation at 94 °C for 30 s, annealing at 57 °C for 1 min 30 s, extension at 72 °C for 1 min, and followed by 30 cycles of denaturation at 94 °C for 30 s, annealing at 52 °C for 1 min 30 s, extension at 72 °C for 1 min, and final extension at 60 °C for 30 min. The size of the PCR products was measured using the ABI PRISM 3130xl Genetic Analyzer (Applied Biosystems) and GENEMAPPER software (Applied Biosystems). PCR and genotyping were replicated 3 to 9 times depending on the genotype observed. The samples that failed to genotype at more than 5 loci were subsequently excluded from the study. Allelic richness (*Ar*) per locus were calculated using HP-Rare 1.1^25^. The number of alleles, expected heterozygosity (*He*), observed heterozygosity (*Ho*), probability of identity (*PID*) and *PID* among siblings (*PID-sib*) were calculated for each species using GenALEx 6.41^26^. Deviation from Hardy–Weinberg Equilibrium (*HWE*) and linkage disequilibrium were tested for using GenALEx 6.41^26^ and GENEPOP ver4.0.10^27^ after Bonferroni correction, respectively.

### Genetic structure analysis

Microsatellite data were also analyzed using the programme STRUCTURE v2.3.3^28^ using the admixture model to estimate population genetic structure and individual ancestries, among and within species. We conducted an analysis with 10 iterations for each population size (*K*) of 1 to 8, and with Markov chain Monte Carlo (MCMC) running for 500,000 generations and initial burn-in of 250,000 generations. The *K* values described by Evanno *et al.*^29^ were then calculated to identify the most reasonable *K* using the programme Structure Harvester^30^. Runs were averaged using CLUMPP version 1. 1. 2^31^, and results were visualized using DISTRUCT version 1. 1^32^ We assessed the average coefficient of membership (*Qi*) of each sampled population to the inferred clusters. Then, we assessed each genotyped to the inferred clusters, based on threshold values of the individual proportion of membership (*qi*). Moreover, the pattern of allelic differentiation between species was explored through Principal Coordinate Analysis (PCoA) by GenALEx 6.41^26^ based on calculated genetic distances.

To test the ability of the markers to identify subspecies (Grant’s zebra and Chapman’s zebra) in the plains zebra, STRUCTURE analysis and PCoA were analyzed in the same way using microsatellite data of plains zebra. To validate the utility of the markers to detect hybridization between Grevy’s zebra and Grant’s zebra (the partially sympatric subspecies), we simulated four hybrid populations (Grevy’s zebra × Grant’s zebra = F1; F1 × F1 = F2; F1 × Grevy’s zebra backcross = BxGy, F1 × Grant’s zebra = BxGt). For the F1 population 20 hybrid individuals were simulated using allele frequencies from Grevy’s zebra and Grant’s zebra with the software HYBRIDLAB^33^. F2 and the two backcross populations (*n* = 20) were developed from the two pure species and the simulated F1 population. We performed STRUCTURE analysis and PCoA as described above using all six populations (Grevy’s zebra, Grant’s zebra, and the four simulated hybrid populations).

## Additional Information

**How to cite this article**: Ito, H. *et al.* Population genetic diversity and hybrid detection in captive zebras. *Sci. Rep.* **5**, 13171; doi: 10.1038/srep13171 (2015).

## Supplementary Material

Supplementary Information

## Figures and Tables

**Table 1:** Results of cross-species amplification performed over the 28 microsatellite loci on the three zebra species and two subspecies of plains zebra (Grant’s zebra and Chapman’s zebra).

	***n***	**Number of genotyped individuals**	**Number of polymorphic loci**	***Ar***	***Na***	***Ho***	***He***	***PID***	***PID-sib***
Grevy’s zebra	60	52	25	2.44 (1.00–4.23)	4.07 (1–9)	0.403 (0.000–0.885)	0.427 (0.000–0.782)	2.28 × 10^−14^	1.06 × 10^−6^
Plains zebra	53	27	27	3.21 (1.00–4.97)	5.32 (1–9)	0.496 (0.000–0.889)	0.573 (0.000–0.829)	7.63 × 10^−21^	5.67 × 10^−9^
Mountain zebra	10	6	23	2.47 (1.00–5.24)	2.75 (1–7)	0.305 (0.000–0.750)	0.368 (0.000–0.764)	7.18 × 10^−20^	1.08 × 10^−8^
Grant’s zebra	33	17	27	3.15 (1.00–5.32)	4.64 (1–9)	0.550 (0.000–1.000)	0.559 (0.000–0.847)	3.53 × 10^−18^	3.36 × 10^−8^
Chapman’s zebra	20	12	26	2.97 (1.00–4.79)	3.96 (1–7)	0.421 (0.000–0.833)	0.529 (0.000–0.799)	7.43 × 10^−12^	9.77 × 10^−6^

Significance of deviation from Hardy–Weinberg equilibrium at P-levels 0.05 (*) and 0.01 (**), and Bonferroni corrected

Abbreviations: *n*; number of tested individuals, *Ar*, allelic richness; *Na*, observed no. of alleles; *Ho*, observed heterozygosity; *He* expected heterozygosity; *PID*, Probability of Identity (unrelated); *PID-sib*, Probability of Identity of siblings.

## References

[b1] Novellie P. Equus zebra, The IUCN Red List of Threatened Species, (2008) Available at: http://www.iucnredlist.org. (Accessed: 23rd June 2015).

[b2] Hack M. A. & Lorenzen E. Equus quagga, The IUCN Red List of Threatened Species, (2008) Available at: http://www.iucnredlist.org. (Accessed: 23rd June 2015).

[b3] Moehlman P. D., Rubenstein D. I. & Kebede F. Equus grevyi, The IUCN Red List of Threatened Species, (2013) Available at: http://www.iucnredlist.org. (Accessed: 23rd June 2015).

[b4] Moodley Y. & Harley E. H. Population structuring in mountain zebras (*Equus zebra*): The molecular consequences of divergent demographic histories. Conserv. Gnenet. 6, 953–968, 10.1007/s10592-005-9083-8 (2006).

[b5] Lorenzen E. D., Arctander P. & Siegismund H. R. High variation and very low differentiation in wide ranging plains zebra (*Equus quagga*): insights from mtDNA and microsatellites. Mol. Ecol. 17, 2812–2824, 10.1111/j.1365-294X.2008.03781.x (2008). 18466230

[b6] Ogden R., Langenhorst T., McEwing R. & Woodfine T. Genetic markers and sample types for pedigree reconstruction in Grevy’s zebra (*Equus grevyi*). Der Zoologische Garten 77, 29–35, 10.1016/j.zoolgart.2007.06.001 (2007).

[b7] Cordingley J. E. *et al.* Is the endangered Grevy’s zebra threatened by hybridization? Anim. Conserv. 12, 505–513, 10.1111/j.1469-1795.2009.00294.x (2009).

[b8] Guichoux E. *et al.* Current trends in microsatellite genotyping. Mol. Ecol. Resour. 11, 591–611, 10.1111/j.1755-0998.2011.03014.x (2011). 21565126

[b9] Ruiz-Rodriguez C. T., Ishida Y., Greenwood A. D. & Roca A. L. Development of 14 microsatellite markers in the Queensland koala (*Phascolarctos cinereus adustus*) using next generation sequencing technology. Conserv. Genet. Resour. 6, 429–431, 10.1007/s12686-013-0115-2 (2014). 25067980 PMC4109682

[b10] Zou H., Dong H., Kong W., Ma J. & Liu J. Characterization of 18 polymorphic microsatellite loci in the red-crowned crane (*Grus japonensis*), an endangered bird. Anim. Sci. J. 81, 519–522, 10.1111/j.1740-0929.2010.00779.x (2010). 20662824

[b11] Yu J. N., Won C., Jun J., Lim Y. & Kwak M. Fast and cost-effective mining of microsatellite markers using NGS technology: an example of a Korean water deer *Hydropotes inermis argyropus*. PLoS One 6, e26933, 10.1371/journal.pone.0026933 (2011). 22069476 PMC3206051

[b12] Miller A. D., Good R. T., Coleman R. A., Lancaster M. L. & Weeks A. R. Microsatellite loci and the complete mitochondrial DNA sequence characterized through next generation sequencing and *de novo* genome assembly for the critically endangered orange-bellied parrot, Neophema chrysogaster. Mol. Biol. Rep. 40, 35–42, 10.1007/s11033-012-1950-z (2013). 23114913

[b13] Santure A. W., Gratten J., Mossman J. A., Sheldon B. C. & Slate J. Characterisation of the transcriptome of a wild great tit *Parus major* population by next generation sequencing. BMC Genomics 12, 283, 10.1186/1471-2164-12-283 (2011). 21635727 PMC3125266

[b14] Ito H., Hayano A., Langenhorst T., Sakamoto H. & Inoue-Murayama M. Using next generation sequencing to develop microsatellite markers for the endangered Grevy’s zebra (*Equus grevyi*). Conserv. Genet. Resour. 5, 507–510, 10.1007/s12686-012-9839-7 (2013).

[b15] Khosravi R., Rezaei H. R. & Kaboli M. Detecting hybridization between Iranian wild wolf (Canis lupus pallipes) and free-ranging domestic dog (*Canis familiaris*) by analysis of microsatellite markers. Zoolog. Sci. 30, 27–34, 10.2108/zsj.30.27 (2013). 23317363

[b16] Ramadan S. Evaluation of genetic diversity and conservation priorities for Egyptian chickens. Open Journal of Animal Sciences 02, 183–190, 10.4236/ojas.2012.23025 (2012).

[b17] Randi E. Detecting hybridization between wild species and their domesticated relatives. Mol. Ecol. 17, 285–293, 10.1111/j.1365-294X.2007.03417.x (2008). 18173502

[b18] Scandura M., Iacolina L., Apollonio M., Dessì-Fulgheri F. & Baratti M. Current status of the Sardinian partridge (*Alectoris barbara*) assessed by molecular markers. Eur. J. Wildl. Res. 56, 33–42, 10.1007/s10344-009-0286-z (2009).

[b19] Sanz N., Araguas R. M., Fernández R., Vera M. & García-Marín J.-L. Efficiency of markers and methods for detecting hybrids and introgression in stocked populations. Conserv. Gnenet. 10, 225–236, 10.1007/s10592-008-9550-0 (2008).

[b20] Barilani M. *et al.* Detecting introgressive hybridisation in rock partridge populations (*Alectoris graeca*) in Greece through Bayesian admixture analyses of multilocus genotypes. Conserv. Gnenet. 8, 343–354, 10.1007/s10592-006-9174-1 (2006).

[b21] Negri A. *et al.* Mitochondrial DNA and microsatellite markers evidence a different pattern of hybridization in red-legged partridge (*Alectoris rufa*) populations from NW Italy. Eur. J. Wildl. Res. 59, 407–419, 10.1007/s10344-012-0686-3 (2012).

[b22] Barilani M. *et al.* Hybridisation with introduced chukars (*Alectoris chukar*) threatens the gene pool integrity of native rock (*A. graeca*) and red-legged (*A. rufa*) partridge populations. Biol. Conserv. 137, 57–69, 10.1016/j.biocon.2007.01.014 (2007).

[b23] Faircloth B. C. Msatcommander: detection of microsatellite repeat arrays and automated, locus-specific primer design. Mol. Ecol. Resour. 8, 92–94, 10.1111/j.1471-8286.2007.01884.x (2008). 21585724

[b24] Rozen S. & Skaletsky H. Primer3 on the WWW for general users and for biologist programmers. Methods Mol. Biol. 132, 365–386 (2000). 10547847 10.1385/1-59259-192-2:365

[b25] Kalinowski S. T. hp-rare 1.0: a computer program for performing rarefaction on measures of allelic richness. Mol. Ecol. Notes 5, 187–189, 10.1111/j.1471-8286.2004.00845.x (2005).

[b26] Peakall R. & Smouse P. E. GenAlEx 6.5: genetic analysis in Excel. Population genetic software for teaching and research—an update. Bioinformatics 28, 2537–2539, 10.1093/bioinformatics/bts460 (2012). 22820204 PMC3463245

[b27] Rousset F. genepop'007: a complete re-implementation of the genepop software for Windows and Linux. Mol. Ecol. Resour. 8, 103–106, 10.1111/j.1471-8286.2007.01931.x (2008). 21585727

[b28] Pritchard J. K., Stephens M. & Donnelly P. Inference of population structure using multilocus genotype data. Genetics 155, 945–959 (2000). 10835412 10.1093/genetics/155.2.945 PMC1461096

[b29] Evanno G., Regnaut S. & Goudet J. Detecting the number of clusters of individuals using the software STRUCTURE: a simulation study. Mol. Ecol. 14, 2611–2620, 10.1111/j.1365-294X.2005.02553.x (2005). 15969739

[b30] Earl D. & vonHoldt B. STRUCTURE HARVESTER: a website and program for visualizing STRUCTURE output and implementing the Evanno method. Conserv. Genet. Resour. 4, 359–361, 10.1007/s12686-011-9548-7 (2012).

[b31] Jakobsson M. & Rosenberg N. A. CLUMPP: a cluster matching and permutation program for dealing with label switching and multimodality in analysis of population structure. Bioinformatics 23, 1801–1806, 10.1093/bioinformatics/btm233 (2007). 17485429

[b32] Rosenberg N. A. Distruct: a program for the graphical display of population structure. Mol. Ecol. Notes 4, 137–138, 10.1046/j.1471-8286.2003.00566.x (2004).

[b33] Nielsen E. E., Bach L. A. & Kotlicki P. Hybridlab (version 1.0): a program for generating simulated hybrids from population samples. Mol. Ecol. Notes 6, 971–973, 10.1111/j.1471-8286.2006.01433.x (2006).

