# Supplementary material for: Population genetic diversity and hybrid detection in captive zebras
Source: Sci Rep. 2015 Aug 21;5:13171. doi: 10.1038/srep13171 (PMC4544005; doi:10.1038/srep13171)
Supplement: Supplementary Information [file srep13171-s1.pdf]

Supplementary Information

Population genetic diversity and hybrid detection in captive zebras

Hideyuki Ito, Tanya Langenhorst, Rob Ogden, Miho Inoue-Murayama

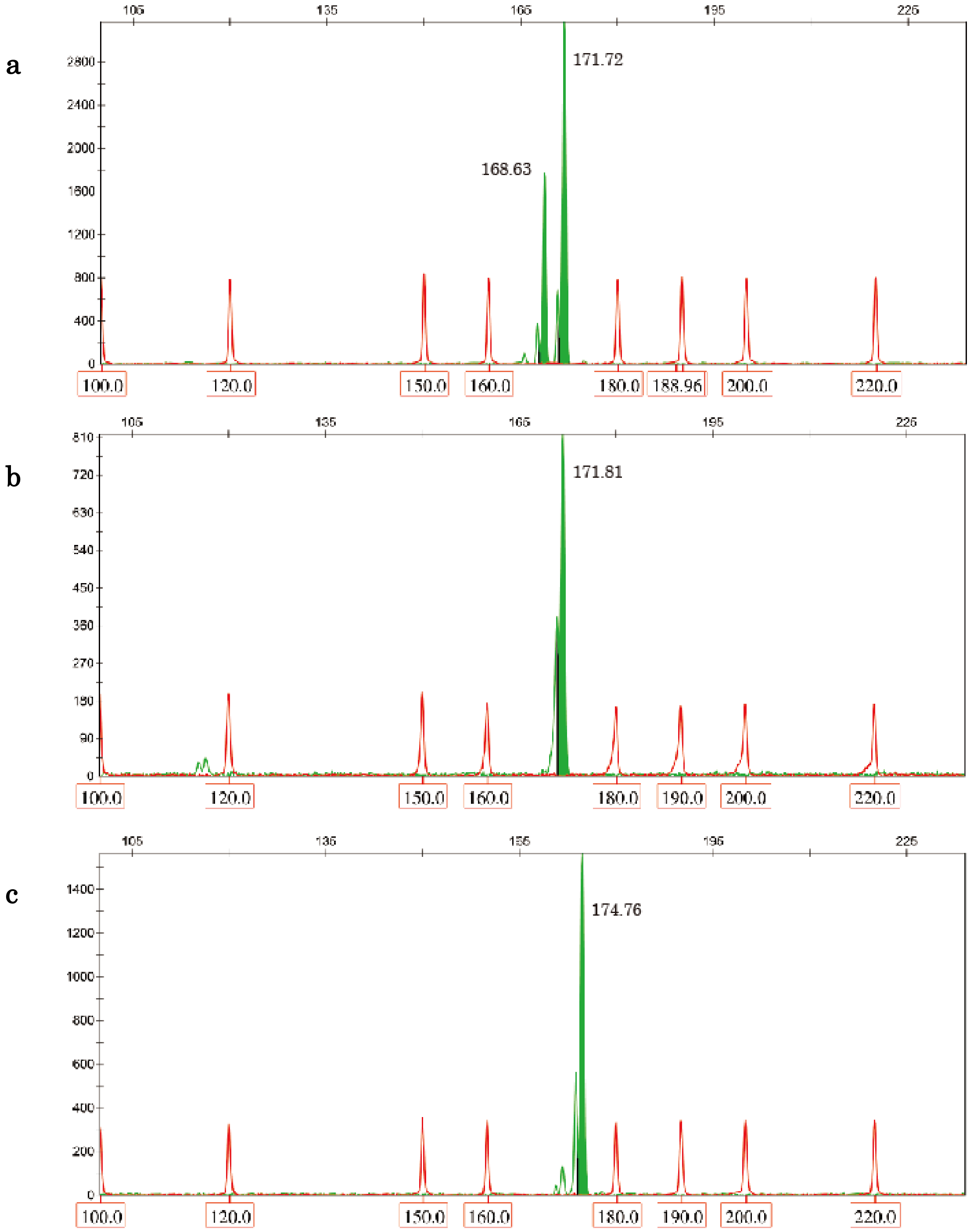

Supplementary Figure S1 Example of peak patterns of locus *EGR02*. (a) *169/172* heterozygote, (b) *172/172* homozygote, (c) *175/175* homozygote.

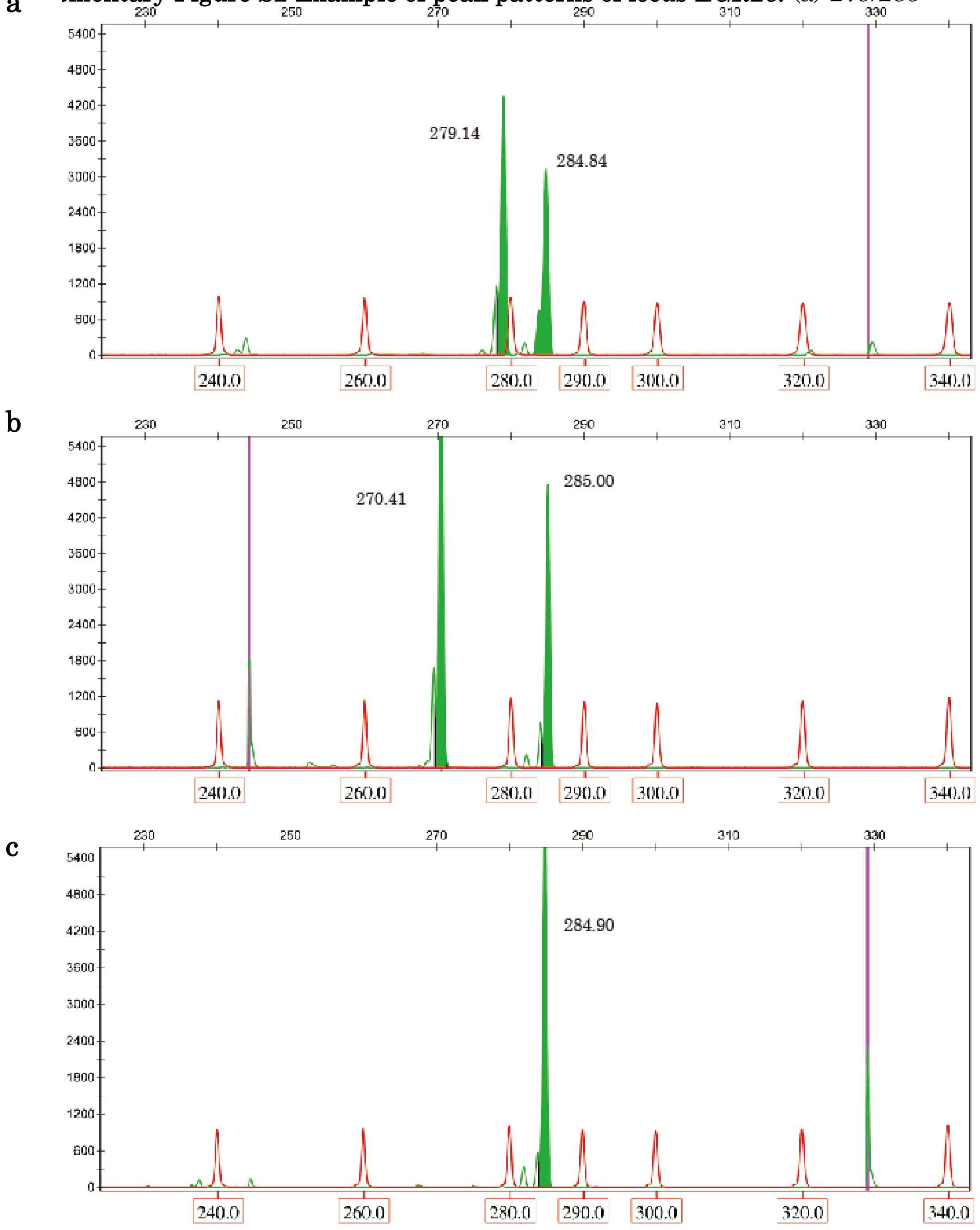

ementary Figure S2 Example of peak patterns of locus *EGR26*. (a) *279/285* heterozygote, (b) *270/285* heterozygote, (c) *285/285* homozygote.

Supplementary Table S1 Information and summary statistics for 28 microsatellite markers in three zebra species

			Grevy's zebra (*Equus grevyi*)							Plains zebra (*Equus quagga*)							Mountain zebra (*Equus zebra*)						
Locus	Repeat unit	Allele length	*n*	*Ar*	*Na*	*Ho*	*He*	*PID*	*PID-sib*	*n*	*Ar*	*Na*	*Ho*	*He*	*PID*	*PID-sib*	*n*	*Ar*	*Na*	*Ho*	*He*	*PID*	*PID-sib*
EGR01	$(CAG)_5$	343-346	52	1.79	2	0.231	0.286	0.550	0.744	26	1.29	2	0.000	0.074**	0.860	0.928	6	1.00	1	0.000	0.000	1.000	1.000
EGR02	$(AAT)_{11}$	163-180	50	2.35	4	0.440	0.521	0.321	0.570	27	2.33	4	0.296	0.445	0.367	0.619	6	1.67	2	0.167	0.153	0.729	0.856
EGR03	$(AGG)_8$	304-322	52	1.99	2	0.365	0.500	0.375	0.594	27	3.10	5	0.667	0.647	0.191	0.474	6	1.00	1	0.000	0.000	1.000	1.000
EGR05	$(CTT)_9$	154-175	52	2.70	5	0.558	0.595	0.247	0.514	27	3.12	6	0.667	0.638	0.197	0.480	6	3.00	4	0.500	0.417	0.364	0.633
EGR07	$(GGT)_8$	238-250	51	2.33	3	0.510	0.544	0.312	0.556	26	3.40	5	0.423	0.618	0.181	0.486	6	1.91	2	0.000	0.278	0.560	0.751
EGR08	$(AGAT)_{10}$	218-245	51	3.44	5	0.549	0.686	0.145	0.443	27	3.78	7	0.704	0.718	0.125	0.422	6	2.67	3	0.667	0.569	0.278	0.535
EGR09	$(AGAT)_{11}$	379-403	51	3.01	4	0.627	0.642	0.191	0.477	26	4.14	6	0.654	0.755	0.094	0.396	5	2.98	3	0.200	0.620	0.217	0.494
EGR10	$(AGGC)_8$	336-363	51	1.92	5	0.294	0.260	0.565	0.761	26	2.71	6	0.538	0.442	0.337	0.613	6	2.58	3	0.500	0.403	0.396	0.648
EGR11	$(ATCT)_{11}$	315-335	52	3.01	4	0.654	0.595	0.215	0.506	26	3.74	6	0.538	0.705	0.126	0.429	5	1.80	2	0.200	0.180	0.689	0.832
EGR12	$(ATCT)_9$	301-330	52	3.91	6	0.712	0.759	0.098	0.395	27	4.15	8	0.556	0.757	0.094	0.395	6	4.15	5	0.500	0.681	0.140	0.445
EGR13	$(ATCT)_9$	249-266	52	2.90	4	0.538	0.616	0.220	0.497	27	2.30	3	0.593	0.444	0.370	0.621	6	2.66	3	0.333	0.542	0.292	0.552
EGR14	$(CA)_{13}$	213-227	50	2.72	3	0.600	0.575	0.244	0.523	27	3.18	6	0.630	0.643	0.189	0.476	6	2.91	3	0.500	0.625	0.220	0.493
EGR15	$(CA)_{14}$	163-187	52	4.23	8	0.885	0.782	0.080	0.379	24	3.70	6	0.333	0.720**	0.124	0.421	6	3.48	4	0.667	0.597	0.207	0.503
EGR16	$(CA)_{18}$	300-328	52	3.77	8	0.673	0.689	0.129	0.438	27	4.81	7	0.889	0.829	0.051	0.348	6	3.80	4	0.500	0.708	0.135	0.429
EGR18	$(CAG)_4$	295-304	48	1.93	4	0.229	0.245	0.581	0.773	26	1.94	2	0.538	0.393	0.445	0.665	5	1.00	1	0.000	0.000	1.000	1.000
EGR21	$(CAG)_4$	276-303	51	1.45	5	0.078	0.113	0.789	0.891	26	2.15	4	0.038	0.371**	0.441	0.675	6	1.91	2	0.000	0.278	0.560	0.751
EGR22	$(CAG)_4$	171-188	52	1.15	3	0.038	0.038	0.926	0.963	20	1.00	1	0.000	0.000	1.000	1.000	5	2.60	3	0.200	0.340	0.461	0.695
EGR24	$(AAC)_{10}$	202-219	52	1.39	2	0.115	0.109	0.800	0.896	26	2.53	4	0.462	0.544	0.291	0.551	6	1.00	1	0.000	0.000	1.000	1.000
EGR25	$(AAT)_9$	378-393	52	1.00	1	0.000	0.000	1.000	1.000	27	3.79	6	0.593	0.729	0.117	0.415	4	2.00	2	0.000	0.375	0.461	0.678
EGR26	$(ACC)_9$	270-288	52	1.08	2	0.019	0.019	0.962	0.981	27	2.88	5	0.556	0.514	0.274	0.562	5	1.80	2	0.200	0.180	0.689	0.832
EGR27	$(CTT)_9$	142-163	52	1.08	2	0.019	0.019	0.962	0.981	23	4.81	8	0.652	0.819	0.055	0.354	6	1.91	2	0.333	0.278	0.560	0.751
EGR28	$(GAT)_9$	250-262	51	1.00	1	0.000	0.000	1.000	1.000	26	1.71	2	0.269	0.233	0.615	0.787	6	1.91	2	0.000	0.278	0.560	0.751

EGR29	$(GAT)_9$	280-301	51	1.00	1	0.000	0.000	1.000	1.000	26	2.09	4	0.308	0.276	0.536	0.746	5	1.00	1	0.000	0.000	1.000	1.000
EGR30	$(CA)_{13}$	136-172	51	4.12	9	0.784	0.727	0.102	0.412	23	4.97	9	0.739	0.820	0.052	0.353	4	4.00	4	0.750	0.656	0.169	0.464
EGR31	$(CA)_{14}$	367-383	46	3.33	4	0.543	0.666	0.159	0.457	24	3.25	6	0.583	0.601	0.201	0.500	6	2.65	3	0.500	0.486	0.327	0.589
EGR32	$(CA)_{15}$	342-360	52	3.58	5	0.692	0.727	0.121	0.417	26	3.63	5	0.615	0.712	0.129	0.426	4	4.00	4	0.750	0.563	0.229	0.526
EGR33	$(CA)_{16}$	326-348	52	3.41	7	0.692	0.672	0.154	0.453	27	4.77	9	0.407	0.800*	0.061	0.365	6	5.24	7	0.667	0.764	0.080	0.388
EGR34	$(CA)_{16}$	196-216	52	2.68	5	0.442	0.585	0.257	0.522	27	4.57	7	0.630	0.810	0.063	0.361	5	2.60	3	0.400	0.340	0.461	0.695
Average				2.44	4.07	0.403	0.427	0.447	0.648		3.21	5.32	0.496	0.573	0.271	0.531		2.47	2.75	0.305	0.368	0.492	0.689

a: developed previous study [14], b: developed in this study

Significance of deviation from Hardy–Weinberg equilibrium at P-levels 0.05 (*) and 0.01 (**), and Bonferroni corrected

Abbreviations: *n*, number of genotyped individuals, *Ar*, allelic richness; *Na*, observed no. of alleles; *Ho*, observed heterozygosity; *He,* expected heterozygosity; *PID*, Probability of Identity (unrelated); *PID-sib*, Probability of Identity of siblings.

Supplementary Table S2 Information and summary statistics for 28 microsatellite markers in two plains zebra subspecies (Grant's and Chapman's zebras)

Locus	Repeat unit	Grant's zebra (*Equus quagga boehmi*)							Chapman's zebra (*Equus quagga chapmani*)						
		n	*Ar*	*Na*	*Ho*	*He*	*Pid*	*Pid-sib*	*n*	*Ar*	*Na*	*Ho*	*He*	*Pid*	*Pid-sib*
EGR01	$(CAG)_5$	15	1.47	2	0.000	0.124*	0.774	0.881	11	1.00	1	0.000	0.000	1.000	1.000
EGR02	$(AAT)_{11}$	15	2.41	4	0.267	0.420	0.382	0.635	12	2.30	3	0.333	0.469	0.360	0.606
EGR03	$(AGG)_8$	15	2.71	4	0.600	0.562	0.268	0.536	12	3.24	4	0.750	0.677	0.167	0.453
EGR05	$(CTT)_9$	15	2.72	4	0.667	0.576	0.261	0.528	12	3.21	4	0.667	0.674	0.172	0.456
EGR07	$(GGT)_8$	15	2.55	4	0.267	0.429	0.362	0.626	11	4.21	5	0.636	0.760	0.094	0.393
EGR08	$(AGAT)_{10}$	15	3.31	5	0.867	0.662	0.172	0.462	12	3.78	5	0.500	0.701	0.131	0.432
EGR09	$(AGAT)_{11}$	15	3.73	6	0.600	0.693	0.137	0.438	11	4.31	5	0.727	0.781	0.083	0.380
EGR10	$(AGGC)_8$	15	2.89	5	0.600	0.480	0.300	0.585	11	2.49	4	0.455	0.380	0.411	0.663
EGR11	$(ATCT)_{11}$	15	3.71	5	0.667	0.698	0.133	0.434	11	3.88	6	0.364	0.702	0.131	0.431
EGR12	$(ATCT)_9$	15	4.28	6	0.600	0.773	0.086	0.385	12	3.78	6	0.500	0.698	0.140	0.436
EGR13	$(ATCT)_9$	15	1.92	2	0.467	0.358	0.476	0.690	12	2.66	3	0.750	0.531	0.283	0.555
EGR14	$(CA)_{13}$	15	3.69	6	0.667	0.676	0.146	0.449	12	2.00	2	0.583	0.497	0.377	0.596
EGR15	$(CA)_{14}$	15	4.00	6	0.533	0.724	0.113	0.416	9	1.99	2	0.000	0.444	0.407	0.630
EGR16	$(CA)_{18}$	15	4.91	7	0.933	0.829	0.053	0.349	12	4.44	6	0.833	0.781	0.080	0.379
EGR18	$(CAG)_4$	14	1.96	2	0.571	0.408	0.434	0.654	12	1.94	2	0.500	0.375	0.461	0.678
EGR21	$(CAG)_4$	14	1.99	2	0.000	0.459*	0.398	0.620	12	1.67	3	0.083	0.156**	0.718	0.851
EGR22	$(CAG)_4$	11	1.00	1	0.000	0.000	1.000	1.000	9	1.00	1	0.000	0.000	1.000	1.000
EGR24	$(AAC)_{10}$	15	2.72	4	0.600	0.576	0.261	0.528	11	2.34	3	0.273	0.492	0.342	0.590
EGR25	$(AAT)_9$	15	3.65	6	0.800	0.671	0.151	0.452	12	3.72	5	0.333	0.694	0.138	0.437
EGR26	$(ACC)_9$	15	2.61	4	0.600	0.464	0.332	0.601	12	3.12	4	0.500	0.559	0.235	0.529
EGR27	$(CTT)_9$	11	4.84	7	0.818	0.810	0.063	0.361	12	3.79	6	0.500	0.698	0.135	0.435
EGR28	$(GAT)_9$	15	1.87	2	0.400	0.320	0.514	0.718	11	1.36	2	0.091	0.087	0.838	0.916

EGR29	$(GAT)_9$	15	1.94	3	0.267	0.240	0.591	0.778	11	2.13	3	0.364	0.310	0.504	0.721
EGR30	$(CA)_{13}$	15	5.32	9	1.000	0.847	0.041	0.337	8	3.77	6	0.250	0.578	0.203	0.512
EGR31	$(CA)_{14}$	15	3.51	6	0.533	0.600	0.190	0.497	9	2.44	3	0.667	0.537	0.311	0.559
EGR32	$(CA)_{15}$	15	3.49	4	0.600	0.696	0.144	0.438	11	3.45	4	0.636	0.690	0.153	0.443
EGR33	$(CA)_{16}$	15	4.72	8	0.667	0.796	0.068	0.369	12	4.31	6	0.083	0.747**	0.096	0.401
EGR34	$(CA)_{16}$	15	4.24	6	0.800	0.771	0.088	0.386	12	4.79	7	0.417	0.799	0.066	0.367
Average			3.15	4.64	0.550	0.559	0.283	0.541		2.97	3.96	0.421	0.529	0.323	0.566

a: developed previous study [14], b: developed in this study

Significance of deviation from Hardy–Weinberg equilibrium at P-levels 0.05 (*) and 0.01 (**), and Bonferroni corrected

Abbreviations as per Supplementary Table S1.
